# Complementary Role of HCV and HIV in T-Cell Activation and Exhaustion in HIV/HCV Coinfection

**DOI:** 10.1371/journal.pone.0059302

**Published:** 2013-03-15

**Authors:** Thijs Feuth, Joop E. Arends, Justin H. Fransen, Nening M. Nanlohy, Karel J. van Erpecum, Peter D. Siersema, Andy I. M. Hoepelman, Debbie van Baarle

**Affiliations:** 1 Department of Internal Medicine and Infectious Diseases, University Medical Center Utrecht (UMCU), Utrecht, The Netherlands; 2 Department of Immunology, University Medical Center Utrecht (UMCU), Utrecht, The Netherlands; 3 Department of Gastroenterology and Hepatology, University Medical Center Utrecht (UMCU), Utrecht, The Netherlands; University of Toronto, Canada

## Abstract

**Objectives:**

To investigate whether T-cell activation and exhaustion is linked to HCV- and HIV disease parameters in HIV/HCV infected individuals, we studied T-cell characteristics in HIV/HCV coinfected patients and controls.

**Methods:**

14 HIV/HCV coinfected, 19 HCV monoinfected, 10 HIV monoinfected patients and 15 healthy controls were included in this cross-sectional study. Differences in expression of activation and exhaustion markers (HLA-DR, CD38, PD-1, Tim-3 and Fas) and phenotypic markers on CD4^+^ and CD8^+^ T-cells were analysed by flow cytometry and were related to HCV disease parameters (HCV-viremia, ALT and liver fibrosis).

**Results:**

Frequencies of activated CD4^+^ and CD8^+^ T-cells were higher in HIV/HCV-coinfected compared to healthy controls and HCV or HIV mono-infected individuals. Coinfected patients also showed high expression of the exhaustion marker PD-1 and death receptor Fas. In contrast, the exhaustion marker Tim-3 was only elevated in HIV-monoinfected patients. T-cell activation and exhaustion were correlated with HCV-RNA, suggesting that viral antigen influences T-cell activation and exhaustion. Interestingly, increased percentages of effector CD8^+^ T-cells were found in patients with severe (F3–F4) liver fibrosis compared to those with no to minimal fibrosis (F0–F2).

**Conclusions:**

HIV/HCV coinfected patients display a high level of T-cell activation and exhaustion in the peripheral blood. Our data suggest that T-cell activation and exhaustion are influenced by the level of HCV viremia. Furthermore, high percentages of cytotoxic/effector CD8^+^ T-cells are associated with liver fibrosis in both HCV monoinfected and HIV/HCV coinfected patients.

## Introduction

Co-infection with human immunodeficiency virus (HIV) is relatively common in hepatitis C virus (HCV) infected patients because of shared routes of viral transmission. [1] HIV/HCV-coinfection is associated with an accelerated course of HCV disease progression and increased HCV viral loads compared to HCV-monoinfection, even when HIV is effectively treated.[1]–[3] Several factors may contribute to this poor prognosis in coinfected patients. Reduced HCV-specific T cell responses have been demonstrated in coinfected patients in the chronic stage of HCV infection, but these studies were limited by either analyzing data of interferon-γ producing cells only [4] or by describing a rather heterogeneous study population including untreated HIV as well as patients on antiretroviral treatment.[4]–[6] A recent study, investigating the production of interferon-γ (IFN-γ) and tumor necrosis factor-α (TNF-α), found similar HCV-specific T-cell responses in HIV-HCV co-infected patients on antiretroviral treatment compared to HCV monoinfected individuals. [7] Other factors possibly contributing to disease progression in HIV/HCV-coinfection include reduced CD4^+^ T-cell help in elimination of infected hepatocytes and direct or indirect cytopathic effects of HIV. [8] Increased immune activation has also been proposed as one of the underlying mechanisms of poor clinical outcome of HCV infection in HIV/HCV-coinfected patients. [9].

Next to generalised T cell activation, chronic viral infection is associated with loss of effector and proliferative functions of CD8^+^ T cells, leading to ineffective viral control. [10] Among other markers of this so-called immune exhaustion, an important function of programmed death receptor 1 (PD-1) has been reported in both HIV and HCV infection and blockage of PD-1 has proved to restore immune function in chronic infection.[10]–[12] Furthermore, dual expression of exhaustion markers Tim-3 and PD-1 on HCV-specific T cells was shown to be correlated with disease progression in HIV-HCV coinfected patients. [13].

We have previously shown increased expression of the death receptor Fas (CD95) on peripheral CD4+ and CD8+ T-cells in chronic HCV infected patients. [14] This could be a sign of immune activation in these patients similar to the observations of increased immune activation in HIV-patients on effective HAART. [15], [16] However, little is known about the additive effect of co-infection with HCV on immune activation in HIV-infected individuals on HAART. A few studies have examined T-cell activation and exhaustion in HIV/HCV co-infection, most of them either lacking a HIV-positive control group or being performed on frozen samples. [5], [17], [18] To study the contributions of HIV and HCV on T cell activation and exhaustion, we used freshly obtained blood to characterize T-cell phenotypes, activation and exhaustion in HIV/HCV-coinfected patients compared to control groups of healthy individuals, HCV-monoinfected and HIV-monoinfected patients. Additionally, we investigated correlations of T-cell phenotype with HCV disease parameters including stage of liver fibrosis, level of HCV viremia, level of alanine transaminase (ALT) to unravel the contributions of these factors to immune activation.

In the present study we demonstrate that T cell activation and exhaustion are increased in patients with HIV/HCV coinfection compared to control groups. In addition, T-cell activation and exhaustion are correlated with the level of HCV-RNA, suggesting that viral antigen drives T cell activation and exhaustion.

## Methods

### Ethics Statement

Informed consent was obtained in writing from all patients in accordance with the WMA Declaration of Helsinki and in accordance with the ICH guideline for Good Clinical Practice (6th revision, 2008). The medical ethics committee for research in humans (METC) of the University Medical Center Utrecht, The Netherlands, approved the protocol of this study.

### Patients

A total of 58 subjects, including 19 chronic HCV monoinfected patients, 10 HIV-1 monoinfected patients, 14 HIV/HCV coinfected patients and 15 healthy controls were included in this study. All patients were recruited from the Infectious Diseases outpatient clinic or from the Gastroenterology outpatient clinic of the University Medical Center Utrecht (UMCU). All patients were negative for hepatitis B surface antigen (HBsAg). None of the patients received treatment for HCV at the time of inclusion or within 12 months before. All HIV-infected patients were on highly active antiretroviral therapy (HAART), resulting in CD4-counts >200/µL and undetectable HIV viral load (<50 copies/mL). Patients with other diseases possibly interfering with their immune system (e.g. liver disease from non-viral causes, auto-immune disease, malignity or any other severe systemic diseases) were excluded from the study, as well as patients with known prior or present alcohol abuse. In the HCV-monoinfected and the HIV/HCV-coinfected group, liver fibrosis was assessed using transient elastography (Fibroscan® (FS), www.echosens.com, Paris, France). Classification of fibrosis was done using the METAVIR scoring system with F0–F2 being no to mild fibrosis (cut-off <9.5 kPa) and METAVIR F3–F4 being sever fibrosis to cirrhosis (cut-off >9.5 kPa). In patients with recent assessment of fibrosis by liver biopsy (<1 year before inclusion), or known cirrhosis, no fibroscan was required. Levels of HCV-RNA and HIV viral load were measured with COBAS® AmpliPrep/COBAS® TaqMan Polymerase Chain Reaction (PCR; lower limit of detection 15 IU/mL for HCV and 50 copies/ml for HIV). Blood from anonymous healthy controls was requested from the bloodbank Mini Donor Dienst of the UMC Utrecht and was tested negative for hepatitis B, hepatitis C and HIV. All patients and healthy controls were between 18 and 65 years old.

### Processing of Blood for Isolation of PBMCs and Analysis with Flow Cytometry

From all patients whole blood was collected by vena puncture in sodium heparin tubes (approximately 27 mL) for PBMCs. Within 8 hours, peripheral blood mononuclear cells (PBMCs) were isolated by standard density centrifugation using Ficoll Hypaque. Per patient, 5 million freshly isolated PBMCs were washed twice with phosphate buffered saline (PBS) and directly stained for markers of T-cell phenotype, activation and exhaustion. The following antibodies were used: anti-CD3 (label: V500; provided by: BD horizon; clone: SP34-2), CD4 (eFluor780; eBioscience; RPA-T4 and PE-Cy7; BioLegend; L3T4), CD8 (PB, BioLegend; RPA-T8 and eFluor780; eBioscience; RPA-T8), CD27 (eFluor780; eBioscience; O323); CD38 (R-PE; Invitrogen; HIT2); CD45RO (APC; BioLegend; UCHL1); CD95 (APC; BP Pharmigen; DX2); PD-1 (PerCP/Cy5.5; BioLegend; EH12.2H7); Tim3 (PE, BioLegend; F38-2E2). Cells were incubated with the antibodies for 20 minutes at 4°C. After washing with PBS/0.5% bovine serum albumin, cells were fixed with Cellfix (BD) and directly analysed by flow cytometry. As an additional marker for effector T-cells, we analysed intracellular perforin expression. To this end, freshly isolated PBMCs were, directly after staining of surface markers, permeabilized and lysed (FACS permeabilizing solution 2 and FACS lysis solution; BD). After permeabilization, cells were incubated with anti-perforin (FITC; δG9; BD). After washing, cells were fixed with Cellfix (BD) and directly analysed on an LSR II FACS machine (BD). Per sample, 100.000 events were acquired. This resulted in exclusion of 2 healthy controls, 4 HCV-monoinfected and 2 HIV/HCV-coinfected patients for the perforin-staining.

### Statistical Analysis

Medians were compared with Mann Whitney test or, in case of multiple groups, with one-way ANOVA followed by Dunnett’s multiple comparison test. Two-way ANOVA was used for comparing medians with two independent variables. Fisher’s exact tests were used to test relation of categorical variables. Dependence of variables was tested using Spearman’s one-tailed correlation coefficient. Statistical analysis was performed with IBM SPSS Statistics version 19.0 (SPSS Inc., IBM) and GraphPad Prism 5 for Windows version 5.03 (GraphPad Software, Inc).

## Results

### Patient Characteristics

Peripheral blood was drawn from a total of 58 subjects, consisting of 14 HIV/HCV-coinfected patients, 19 HCV-monoinfected patients, 10 HIV-monoinfected patients and 15 uninfected healthy controls. Characteristics of the different patient groups are shown in table 1. None of the patients received treatment for HCV at the time of inclusion. However, eight out of nineteen HCV monoinfected patients (42%) and three out of thirteen HIV/HCV coinfected patients (21%) had a history of ineffective HCV treatment more than 1 year before inclusion, while all others were treatment-naïve (p = 0.07). All HIV-infected patients (both monoinfected and HCV-coinfected) were on HAART at the time of this study, resulting in CD4^+^ T-cell counts above 200 cells/mm^3^ and HIV-RNA <50 copies/mL. Median duration of HAART was 8 years in the monoinfected and 13 years in the coinfected group (p = 0.35). As expected, the ALT value was lower in HIV mono-infected patients (30 IU/L) compared to the HCV-infected patients (65 IU/L; p<0.05), but ALT levels were similar between HIV/HCV coinfected (65 IU/L) and HCV monoinfected patients (69 IU/L; p = 0.92). There was a trend towards higher level of HCV-viremia in coinfected patients (1.9e6 IU/mL) compared to HCV monoinfected patients (4.5e5 IU/mL; p = 0.07) (table 1).

**Table 1:** Patient characteristics.

	HCV/HIV	HCV	HIV	
	n = 14	n = 19	n = 10	P-value
**General characteristics**				
Age, median (IQR), years	48 (14)	55 (7)	46 (6)	0.12
Gender, % male	86%	74%	90%	0.27
ALT, median (IQR), IU/L	65 (80)	69 (85)	30 (22)	<0.001
**HCV disease characteristics**				
HCV-RNA, median (IQR), IU/mL	1.9e6 (4.8e6)	4.5e5 (1.1e6)		0.07
Genotype, % genotype 1	75%	79%		1.00
**History of HCV treatment:**				0.07
Naïve, number (%)	3 (21%)	11 (58%)		
Previously failed treatment, n (%)	11 (79%)	8 (42%)		
**Liver fibrosis/cirrhosis**				0.73
F0–F2, number (%)	8 (57%)	9 (47%)		
F3–F4, number (%)	6 (43%)	10 (53%)		
**HIV disease characteristics**				
Viral load, copies/mL	All <50		All <50	–
CD4-count, median (IQR),/mm^3^	540 (330)		661 (247)	0.11
HAART regimen				0.70
PI-based	7 (50%)		4 (40%)	
Other regimens	7 (50%)		6 (60%)	
Years of HAART, median	13		8	0.35

General and disease-specific characteristics of patient groups. Abbreviations: **ALT**, alanine transaminase; **IQR**, interquartile range; **PI**, protease inhibitor; **HAART**, highly active antiretroviral treatment.

### HIV/HCV Coinfection is Associated with Increased T-cell Activation and PD-1 Expression

**Figure 1:**
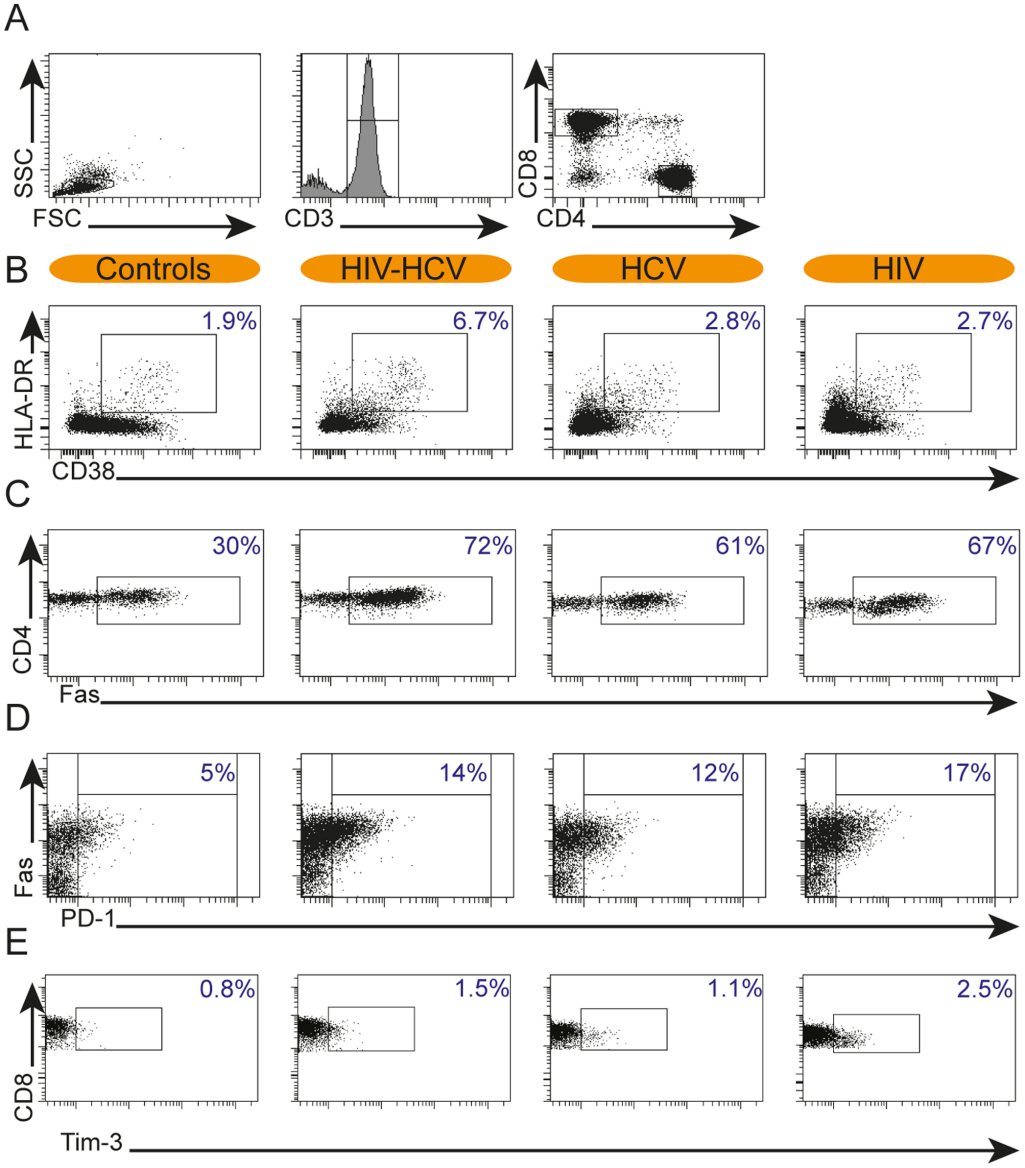
Representative flow cytometry plots. Representative flow cytometry plots of T-cell activation- and exhaustion markers in HIV-HCV coinfected patients, HCV mono- and HIV mono-infected patients and healthy controls. A: gating of CD4^+^ and CD8^+^ T-cells by lymphocyte-gate (left panel), CD3-gate (middle panel) and gates for CD4^+^ or CD8^+^ T-cells (right panel). B-E: representative flow cytometry plots of a healthy control (left), HIV-HCV coinfected (middle left), HCV monoinfected (middle right) and HIV -monoinfected patient (right) showing (B) activated CD8^+^ T-cells; (C) Fas-positive CD4^+^ T-cells; (D) PD-1 positive CD4^+^ T-cells and (E) Tim-3 positive CD8^+^ T cells. Percentages are depicted in the right upper corner.

**Figure 2:**
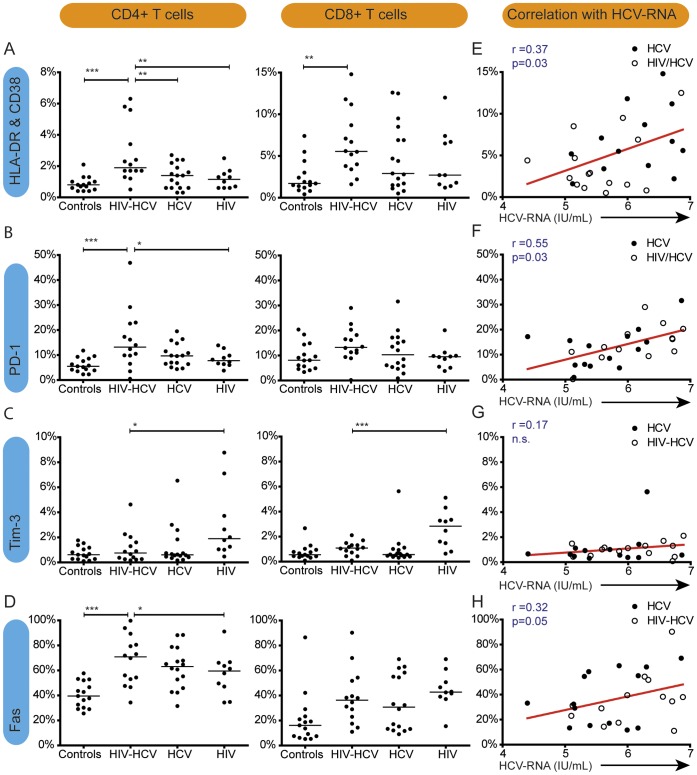
T-cell activation and exhaustion and its correlations with HCV-RNA in HIV/HCV-coinfection and control groups. A: percentages of HLA-DR^+^CD38^+^ activated CD4^+^ (left) and CD8^+^ T-cells (right). B: percentages of PD-1 positive CD4^+^ and CD8^+^ T-cells. C: percentages of Tim3-positive CD4^+^ and CD8^+^ T-cells. D: percentage of death receptor Fas positive CD4^+^ and CD8^+^ T cells. E-H: correlations of HCV viral load within HIV-HCV coinfected (open dots) and HCV monoinfected patients (closed dots) with percentages of HLA-DR and CD38 positive CD8 T cells (E); PD-1 positive CD8 T cells (F); Tim-3 positive CD8 T cells (G) and Fas positive CD8 T cells (H). HCV viral loads are depicted in IU/mL. P-values are indicated with * (p<0.05), ** (p<0.01) or *** (p<0.0001). Spearman r and p-value of correlations are depicted in the upper left corner of each graph; red lines represent semilog fit lines.

To study T-cell activation and exhaustion in HIV/HCV-coinfected patients, we measured expression of activation markers CD38 and HLA-DR [19], exhaustion markers Programmed Death Receptor-1 (PD-1) and T-cell immunoglobulin domain and mucin domain 3 (Tim-3) and death receptor Fas (CD95) in coinfected patients, HIV- or HCV-monoinfected patients and uninfected healthy controls. Gating strategy and representative plots of a healthy control, HCV-monoinfected, HIV-monoinfected and HIV/HCV-coinfected patient are shown in figure 1A–E. Frequencies of activated CD4^+^ and CD8^+^ T-cells, defined by CD38/HLA-DR double positivity, were higher in HIV/HCV-coinfected patients (median 1.9% and 5.6% respectively) compared to healthy controls (medians 0.8%; p<0.001 and 1.7%; p<0.01). Coinfected patients also displayed higher CD4^+^ T-cell activation (1.9%) in comparison to HCV- or HIV-monoinfected patients (1.4%, p<0.01 and 1.2%, p<0.01) (Fig. 2A). Expression of the T-cell exhaustion marker PD-1 was higher on CD4^+^ T-cells of coinfected patients (13.2%) in comparison with HIV-monoinfected patients (7.8%; p<0.05) and healthy controls (5.5%; p<0.001). A similar pattern of PD-1 expression was observed in CD8^+^ T cells (HIV/HCV: 13.3%, healthy controls: 8.1%, HCV: 10.3%, HIV 9.5%; not significant) (Fig. 2B). Interestingly, Tim-3 expression was not significantly increased in HIV/HCV co-infected patients. In contrast, HIV-monoinfected patients depicted significantly higher levels of Tim3 on both CD4^+^ and CD8^+^ T cells (1.9% and 2.8%) compared to HIV/HCV coinfected patients (0.8%; p<0.05 and 1.1%; p<0.01) and healthy controls (0.6%; p<0.01 and 0.6%; p<0.001) (fig 2C). Numbers of PD-1 and Tim-3 dual positive T cells were too low for reliable analysis.

Furthermore, with high activation and exhaustion in HIV/HCV coinfected patients compared to healthy controls, a higher susceptibility to apoptosis was expected. [20] Indeed, expression of the death receptor Fas on CD4^+^ T-cells of coinfected patients (median 71%) was significantly increased compared to healthy controls (40%, p<0.001) and HIV-infected controls (60%; p<0.05). However, in coinfected patients Fas-expression on CD4^+^ T-cells (71%) did not differ in comparison to HCV monoinfected controls (63%). On CD8^+^ T-cells, there were no significant differences of Fas expression in coinfected patients (36%) compared to HCV or HIV monoinfected patients (31% and 43%) or healthy controls (16%) (Fig. 2B).

### T-cell Activation and Exhaustion are Linked to Level of HCV-RNA in HIV-HCV Co-infection

To elucidate which clinical parameters may contribute to T-cell activation and exhaustion, we investigated correlations of ALT and HCV viremia with expression of HLA-DR/CD38, PD-1, Tim-3, and Fas.

Within all HCV-infected patients, HCV-RNA correlated positively with T cell activation (CD8: r = 0.37, p<0.05; CD4: r = 0.28, p = 0.08) as well as with expression of the exhaustion marker PD-1 (CD4^+^: r = 0.52, p<0.01 and CD8^+^: r = 0.55, p<0.001), whereas there was no correlation with expression of Tim-3. The positive correlation of PD-1 expression was still present when the patients were divided in HCV-monoinfected (CD4: r = 0.49, p = 0.04; CD8: r = 0.39, p = 0.08) and HIV/HCV coinfected patients (CD4∶0.26, not significant; CD8∶0.53, p = 0.03). Additionally, a borderline significant correlation of HCV-RNA and death receptor Fas expression on CD8^+^ T cell was found (r = 0.32, p = 0.05) (figure 2; table 2).

**Table 2:** Correlations of clinical parameters and T cell markers.

	CD4			CD8		
	Spearman R		p-value	Spearman R		p-value
**HCV-RNA**						
HLA-DR^+^CD38^+^	0.28		0.08	0.37		0.03
PD1^+^	0.52		<0.01	0.55		0.03
Tim3^+^	0.01		n.s.	0.17		n.s.
Fas^+^	0.20		n.s.	0.32		0.05
**ALT**						
HLA-DR+CD38+	-0.09		n.s.	0.13		n.s.
PD1+	0.16		n.s.	0.26		n.s.
Tim3+	-0.17		n.s.	0.24		n.s.
Fas+	0.24		n.s.	0.12		n.s.
**Liver fibrosis**	**F0–F2**	**F3–F4**	**P-value**	**F0–F2**	**F3–F4**	**p-value**
HLA-DR+CD38+	1.6%	1.9%	n.s.	4.4%	5.4%	n.s.
PD1+	10.2%	10.4%	n.s.	14.6%	9.8%	n.s.
Tim3+	0.6%	1.1%	n.s.	0.8%	0.7%	n.s.
Fas+	57%	66%	n.s.	34%	32%	n.s.

Spearman correlations of HCV-RNA and ALT with expression of activation and exhaustion markers and median expression of these markers in patients with F0–F2 versus F3–F4 fibrosis in all HCV-monoinfected and HIV/HCV-coinfected patients.

In contrast, there was no correlation of T cell activation and exhaustion with ALT (table 2). Furthermore, T-cell activation and exhaustion as well as level of HCV-viremia were similar in patients with F0–F2 fibrosis or F3–F4 fibrosis (table 2). Lastly, a history of failed HCV-treatment was not associated with differences in T-cell activation or exhaustion.

### HCV and HIV are Associated with Differences in Memory and Effector Phenotype of CD8^+^ T-cells

**Figure 3:**
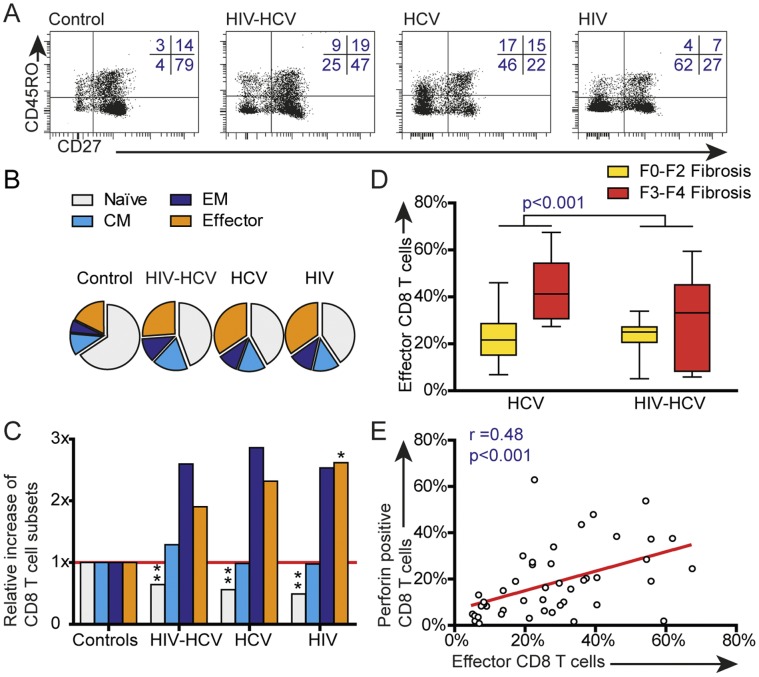
Changes in CD8^+^ effector and memory phenotype in all patients and healthy controls. A: representative plots of a healthy control, HIV-HCV coinfected, HCV monoinfected and HIV- mono patient showing naïve (right lower quadrant), central memory (right upper quadrant), effector memory (left upper quadrant) and effector (left lower quadrant) CD8^+^ T-cells by CD27 and CD45RO staining. B: pie charts of mean percentages of naïve (light grey), central memory (CM; light blue), effector memory (EM; dark blue) and effector (orange) CD8^+^ T-cells in 42 patients and 3 healthy controls. C: relative increase of median percentages of memory subsets compared to healthy controls. D: box plot showing percentages of effector CD8^+^ T-cells in HCV-monoinfected (left) or HIV/HCV-coinfected patients (right) with fibrosis scores F0–F2 (yellow) versus F3–F4 (red). The depicted p-value was calculated with two way ANOVA and indicates statistical significant difference in percentages of effector CD8^+^ T cells in F0–F2 fibrosis compared to F3–F4 fibrosis, independent of coinfection with HIV. Liver fibrosis was assessed with Fibroscan in 74% of HCV monoinfected patients and 71% of HIV/HCV coinfected patients. E: correlation of staining for perforin (Y-axis) with effector phenotype (X-axis) in CD8^+^ T-cells. Box-plots show median, quartiles and range. Line represents linear regression. P-values are depicted as: * (<0.05) and ** (p-value <0.01).

Chronic viral infections are shown to coincide with changes in T-cell memory and effector phenotype. [21] Therefore, we examined whether HIV/HCV-coinfection drives changes in memory and effector phenotype of CD8^+^ T-cells, by studying percentages of naïve (CD45RO^−^CD27^+^), central memory (CD45RO^+^CD27^+^), effector memory (CD45RO^−^CD27^+^) and effector (CD45RO^−^CD27^−^) CD8^+^ T-cells. Representative plots of a healthy control, an HCV monoinfected patient and an HIV/HCV coinfected patient are shown in figure 3A.

Mean percentages of T-cell phenotypes in patients compared to healthy controls are depicted as pie graphs in figure 3B. Due to the small percentages of effector T-cells, changes in these subsets are more apparent when depicted as relative increase compared to healthy controls (Fig. 3C). HIV/HCV coinfected patients showed significantly higher frequencies of central memory CD8^+^ T-cells compared to healthy controls (15.1% versus 11.8% p<0.05). The increase of effector CD8^+^ T-cells was significant in HCV and HIV monoinfection but not in HIV/HCV coinfection (Fig. 3B–C). There was no correlation with levels of HCV-RNA or ALT with memory and effector phenotype.

### Liver Fibrosis is Associated with Increased Frequencies of Effector CD8^+^ T-cells

As T-cell mediated killing of infected hepatocytes is essential in liver fibrogenesis, [22] we investigated whether liver fibrosis was associated with differences in T-cell effector phenotype. Indeed, we found increased effector CD8^+^ T-cell frequencies in patients with F3–F4 liver fibrosis in both HCV monoinfected patients (median 41.3% versus 21.7% in patients with F0–F2 liver fibrosis) and HIV/HCV-coinfected patients (33.2% versus 25.0%; p<0.01) (Fig. 3D). This finding was further confirmed by a positive correlation of percentages effector CD8^+^ T cells with Fibroscan-score (r = 0,57; p = 0.0019) (data not shown). Additional analysis of intracellular perforin expression within CD8^+^ T-cells, as an indicator of effector T-cells with a potency to kill, revealed slightly higher frequencies of perforin-positive CD8^+^ T-cells in mono- and coinfected patients with severe fibrosis, albeit not significant (p = 0.54; data not shown). However, frequencies of perforin-positive CD8^+^ T-cells correlated strongly with effector phenotype (r = 0.48; p<0.001; Fig. 3E). Altogether, these data suggest a role for perforin-positive/effector phenotype CD8^+^ T-cells in liver fibrosis.

## Discussion

HIV/HCV-coinfection is associated with faster HCV disease progression than HCV-monoinfection. [3] The underlying pathogenic mechanisms for poor clinical outcome in HCV/HIV coinfection remain unclear although various mechanisms have been suggested, including increased immune activation. [9], [23] The present study confirms that HIV/HCV coinfected patients on HAART indeed have higher levels of T-cell activation as indicated by CD38/HLA-DR expression. In addition, we show that these T-cells are also high in expression of PD-1 and Fas, both linked to T-cell exhaustion and apoptosis. [24], [25] Furthermore, this study shows correlations of concentration between HCV-RNA and markers for T-cell activation and exhaustion, suggesting a role for HCV viremia in influencing T-cell activation and exhaustion in this group of patients.

Recently, it has become clear that HIV-infected patients on antiretroviral treatment still display slightly increased T-cell activation, [26], [27] despite its initial decrease upon start of antiretroviral treatment. [28] Interestingly, we observed higher T-cell activation in HIV/HCV coinfected patients compared to HIV-monoinfected patients despite longer history of HAART in coinfected patients. [29] Various underlying mechanisms contributing to T-cell activation in those patients have been proposed in literature, of which the concept of microbial translocation is a currently widely accepted model. [16] As we show that the level of T-cell activation correlates with HCV-RNA, we hypothesize that this concomitant viral infection may well contribute to the observed increase of T-cell activation in HIV-infected individuals. Indeed, in a cohort of HCV monoinfected patients [30] we have previously observed a decrease in CD4 and CD8^+^ T cell activation after 4 weeks of IFN-α/ribavirin treatment in the subset of patients with rapid viral response (HCV-RNA <50 IU/mL at week 4 of therapy), whereas there was no change in T cell activation in patients without rapid viral response (unpublished data). Since immune activation is thought to play an important role in long-term morbidity and mortality, [31] it can be hypothesized that coinfection with HCV may contribute to long-term extrahepatic morbidity in HIV-infected individuals through increased immune activation. Indeed, a recent study observed a higher prevalence of subclinical carotid plaque formation in HIV-patients coinfected with HCV compared to HIV-monoinfected patients. [32] However, a prospective study is required to examine whether increased peripheral T-cell activation in HCV-monoinfected and HIV/HCV-coinfected patients is indeed associated with mortality and long-term morbidity.

In this study, T cell exhaustion, measured by PD-1 expression, correlated with HCV-RNA in HCV monoinfected and HIV/HCV coinfected patients. This is in line with a rather old study showing a correlation between HCV viral load and decreased T cell function in HCV-monoinfection, but at that time a role for PD-1 was in T cell exhaustion was not yet discovered. [33] Thus, to our knowledge, we are the first to show increased T cell exhaustion indicated by PD-1 expression is linked to HCV viremia. Based on a study evaluating T cell exhaustion upon LCMV-infection in C57BL/6 mice, [34] it can be hypothesised that high level of viremia contributes to increased exhaustion, although it can also be reasoned that T cell exhaustion by itself may lead to higher antigen levels through decreased viral control. [11] In contrast, Tim-3 was lower in HIV-HCV coinfected patients compared to HIV-monoinfected controls. This finding is supported by data from a recent study by Vali *et al*., in which Tim-3/PD-1 on total CD4^+^ and CD8^+^ T cell pools were highest in HIV-monoinfected patients in comparison with HIV-HCV coinfected and HCV monoinfected patients. [13] Although not significant, our data support the observation by *Vali et al.* that Tim-3 expression is increased in HCV-infected patients in comparison to healthy controls. [13] It must be noted that age and gender of healthy volunteers were not characterised do to the anonymous service of the blood bank.

One possible explanation for decreased Tim-3 expression in HIV-HCV coinfected patients compared to HIV-monoinfected patients could be decreased activity of T-bet, a transcription factor classically associated with Th1 phenotype [35] but recently also shown to induce Tim-3 and repress PD-1 expression. [36], [37] Persistent antigen exposure was shown to lead to downregulation of T-bet. [37] This suggests that downregulation of T-bet due to persistent HCV antigen may be involved in decreased Tim-3 expression and increased PD-1 expression, as we observed in the present study, next to decreased Th1/Th2 ratio, observed by others, [38] associated with coinfection of HCV in HAART-treated HIV patients.

A second interesting observation in our cross-sectional study is the high level of effector CD8^+^ T-cells in HCV monoinfected and HIV/HCV-coinfected patients with severe fibrosis. Few other studies investigate changes of memory and effector T-cell memory and effector compartments in HIV/HCV-coinfection. [5], [17], [18], [39] However, these studies did not compare to HCV- or HIV mono-infected controls or were heterogeneous in terms of HIV treatment, including both patients treated with HAART and patients with untreated HIV, [5], [17], [18], [39] which may result in HIV-antigen driven differences in memory and effector phenotype. To our knowledge, no studies relating these T-cell phenotypes to fibrosis progression have been published. Our data suggests that effector T-cells might play a role in liver fibrosis since it has been shown that similar T-cell populations are present in blood as well as in the liver of HCV infected patients. [40] Killing of infected hepatocytes by intrahepatic cytotoxic T-cells may be perforin-dependent [41] and apoptosis of hepatocytes has a strong profibrotic effect through activation of hepatic stellate cells. [42] We therefore postulate that high frequencies of effector CD8^+^ T-cells may contribute to liver fibrosis by killing infected hepatocytes which in turn activate hepatic stellate cells and promote fibrogenesis. Alternatively, it may be hypothesized that increased percentages in the peripheral blood would reflect an actual decrease at the site of infection due to compartmentalization. However, it has recently been shown that CD27 expression by intrahepatic lymphocytes is reflected by CD27 expression in the peripheral blood. [43] Examination of effector T cell phenotypes in liver biopsies in patients with minimal or severe fibrosis are needed to provide a definite conclusion.

The design of the present study was distinctive for several reasons. First, all stainings for activation and exhaustion markers were performed on freshly isolated PBMCs, thereby eliminating any influence of cryopreservation on expression of activation and exhaustion markers and T-cell phenotype which hampers other studies. [44], [45]. Albeit our sample size is somewhat smaller than other studies, we believe that our data closely reflect the actual phenotype of T-cells in the peripheral blood. Furthermore, patients with no or minimal fibrosis as well as patients with severe fibrosis were included enabling us to investigate whether phenotypic differences are correlated to HCV disease progression. The HIV-infected groups were homogenous, including only patients on successful antiretroviral therapy (HIV not detectable, CD4>200). The frequency of HCV treatment experienced patients was higher in the HCV-monoinfected group in comparison to the HIV/HCV-coinfected group, but a minimum of 1 year interferon free period was required before inclusion and no late effects of interferon are known from literature.

The present study is hampered by the fact that we did not investigate antigen-specific T-cells, we cannot be sure whether our findings with respect to T-cell memory subsets, activation and exhaustion are directly influencing HCV-specific T-cell responses. However, various previous studies have convincingly proved that PD-1 and Tim-3 expression suppress HCV-specific immune responses and thereby contribute to viral persistence in HCV. [46] Furthermore, liver infiltrates are largely composed of HCV-nonspecific T cells, suggesting that disease progression may well be affected by non-specific activation. [40], [47], [48].

From the present study we conclude that T-cells in HIV/HCV-coinfected patients show increased activation and exhaustion surface markers compared to healthy controls as well as to HIV- or HCV (co)infected patients. This suggests that HIV and HCV have a complementary role on T-cell activation in HIV/HCV coinfection. This could at least partly be explained by the level of antigen, since T cell activation and exhaustion correlated with the level of HCV viremia. Furthermore, liver fibrosis is associated with increased frequencies of effector CD8^+^ T-cells, based on extracellular surface markers and intracellular perforin expression, suggesting that these effector CD8^+^ T-cells may contribute to liver fibrosis in a perforin dependent manner.
